# Single-Cell Infection of Influenza A Virus Using Drop-Based Microfluidics

**DOI:** 10.1128/spectrum.00993-22

**Published:** 2022-09-20

**Authors:** Emma Kate Loveday, Humberto S. Sanchez, Mallory M. Thomas, Connie B. Chang

**Affiliations:** a Center for Biofilm Engineering, Montana State University, Bozeman, Montana, USA; b Department of Chemical and Biological Engineering, Montana State University, Bozeman, Montana, USA; c Microbiology and Cell Biology, Montana State University, Bozeman, Montana, USA; d Department of Physiology and Biomedical Engineering, Mayo Clinic, Rochester, Minnesota, USA; Wright State University

**Keywords:** microfluidics, influenza, single cell, drops

## Abstract

Drop-based microfluidics has revolutionized single-cell studies and can be applied toward analyzing tens of thousands to millions of single cells and their products contained within picoliter-sized drops. Drop-based microfluidics can shed insight into single-cell virology, enabling higher-resolution analysis of cellular and viral heterogeneity during viral infection. In this work, individual A549, MDCK, and siat7e cells were infected with influenza A virus (IAV) and encapsulated into 100-μm-size drops. Initial studies of uninfected cells encapsulated in drops demonstrated high cell viability and drop stability. Cell viability of uninfected cells in the drops remained above 75%, and the average drop radii changed by less than 3% following cell encapsulation and incubation over 24 h. Infection parameters were analyzed over 24 h from individually infected cells in drops. The number of IAV viral genomes and infectious viruses released from A549 and MDCK cells in drops was not significantly different from bulk infection as measured by reverse transcriptase quantitative PCR (RT-qPCR) and plaque assay. The application of drop-based microfluidics in this work expands the capacity to propagate IAV viruses and perform high-throughput analyses of individually infected cells.

**IMPORTANCE** Drop-based microfluidics is a cutting-edge tool in single-cell research. Here, we used drop-based microfluidics to encapsulate thousands of individual cells infected with influenza A virus within picoliter-sized drops. Drop stability, cell loading, and cell viability were quantified from three different cell lines that support influenza A virus propagation. Similar levels of viral progeny as determined by RT-qPCR and plaque assay were observed from encapsulated cells in drops compared to bulk culture. This approach enables the ability to propagate influenza A virus from encapsulated cells, allowing for future high-throughput analysis of single host cell interactions in isolated microenvironments over the course of the viral life cycle.

## INTRODUCTION

Single-cell studies of viral infection enable high-resolution examination of heterogeneous virus populations. Differential selective pressures in both the host cell and virus population lead to variability in virus replication and production that enables antiviral escape, zoonotic spillover events, and changes in virulence or pathogenesis (1). Influenza A virus (IAV) is a negative-strand RNA virus with populations containing high genetic diversity due to its segmented genome, rapid replication rate, and low-fidelity RNA-dependent RNA polymerase (RdRp) (1). As such, IAV infection results in a diverse swarm of unique variants exhibiting heterogeneous genotypes and phenotypes (2, 3).

Cutting-edge technologies such as drop-based microfluidics (4–8) and single-cell sequencing (9–14) are enabling higher-resolution analysis of the effects of cellular heterogeneity on viral infection dynamics. Single-cell virology studies have revealed a more detailed snapshot of the heterogeneous kinetics of virus production in relation to innate immune activation and responses (15–18) and the variability of viral gene expression between individually infected cells (9–12). Heterogeneous IAV populations can be examined using methods that capture diversity at the single-cell level. Such methods include microfluidic techniques to perform single-cell transcriptomics (9–11) or isolation of cells using limiting dilutions in well plates to assess single infection events (12, 13, 16, 19) or by using two-dimensional (2D) microfluidic chambers (15). While these studies have identified large variations in total viral mRNA expressed, infectious virus produced, and host transcriptional response to infection, the low-throughput methods were limited to analyzing hundreds to a few thousand individual cells (12, 13, 15, 20). To process tens of thousands or up to millions of single cells, a high-throughput continuous flow method such as fluorescence-activated cell sorting (FACS) can be utilized to study virus infection (9–11, 14). Yet FACS sorting is mostly performed at early time points postinfection to ensure that virus spread is reduced and that the timing of infection, viral gene expression, and cellular response to infection are comparable from cell to cell (9–11, 14, 17). In contrast, analysis of infected cells from bulk cultures at later time points postinfection can be difficult due to virus spread.

A method to study single cells at high throughput is drop-based microfluidics, which offers the ability to compartmentalize and rapidly assay individual cells (6, 21–25). A microfluidic device is used to create microscale aqueous drops that are surrounded by an immiscible oil and stabilized with a biocompatible surfactant (26). Compared to larger-scale *in vitro* culturing methods where cells are grown in flasks or well plates, drop-based microfluidics creates millions of discrete bioreactors that house single cells in which viruses can replicate (4, 7), enabling viral replication events to be analyzed independently. Furthermore, these compartmentalized cells in drops allow for single-cell analysis at any time point postinfection since virus spread cannot occur between neighboring drops. Thus, drops provide a way to analyze virus replication, production, and cellular responses of single cells at multiple time points.

To date, drop-based viral infections have only been performed with murine norovirus (MNV-1), a positive-sense RNA virus in a small icosahedral capsid that lacks an outer envelope and is extremely stable at a range of environmental conditions, using cell lines adapted to spinner cultures or already grown in suspension (4, 7, 8, 27). Drop-based microfluidics was used to study infectivity (4, 7) and recombination (8) of MNV-1, with data demonstrating the ability to successfully encapsulate and infect mammalian cells within drops (4, 6–8). Expanding the applicability of drop-based microfluidics to other viruses, specifically for enveloped viruses such as IAV, and the ability to encapsulate a broad range of host cells would greatly increase capacity for single-cell virology studies.

In this work, drop-based microfluidics was applied toward culturing and studying IAV infections at the single-cell level. Three different cell lines, alveolar basal epithelial (A549) cells, Madin-Darby Canine Kidney (MDCK) cells, and MDCK plus human *siat7e* gene (siat7e) cells (28, 29), were tested for their viability and ability to support IAV infection in drops compared to bulk tissue culture. Drop stability, cell loading, and viability were tested within 100-μm-diameter drops and quantified after 24 h of incubation, sufficient time for one productive round of IAV infection. Additionally, this study demonstrates the use of drop-based microfluidics to culture and propagate enveloped viruses, which generally have reduced environmental stability. The cells were infected with A/California/07/2009 (H1N1) at a multiplicity of infection (MOI) of 0.1 to compare virus production over multiple time points between infected cells on standard tissue culture plates and encapsulated in drops. Our findings demonstrate that standard adherent cell lines can be used in drops to propagate IAV, thereby expanding single-cell capabilities for studying viral infections, which, until recently, has only been demonstrated for nonadherent, spinner-adapted host cells (4, 7, 8).

The drop-based microfluidic methods developed in this work will enable future studies of single-cell IAV infections using multiple cell lines. The methods expand the application of drop-based microfluidics from the nonenveloped positive-sense MNV-1 (4, 7, 8) to include the enveloped and negative-sense IAV, thus broadening the scope of single-cell virology assays. Additionally, this work can serve as a blueprint for testing, comparing, and implementing drop-based microfluidics methods in future single-cell studies. With the recent interest and development of single-cell omic technologies for studying viral infections (13–15, 20, 30–36), we expect this work to further expand capabilities and increase applications of single-cell technologies in virology.

## RESULTS

### Single-cell virus infection using drop-based microfluidics.

**FIG 1:**
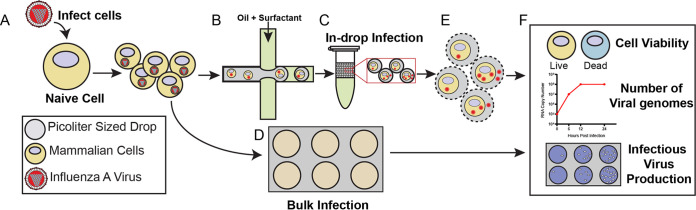
Graphical workflow. (A) Cells were infected with IAV. (B) A suspension of infected cells was encapsulated into drops using a fluorinated oil continuous phase. (C) Drops were incubated for 24 h at 37°C and 5% CO_2_. (D) A portion of infected cells was replated onto standard tissue culture dishes to recapitulate a bulk infection and incubated for 24 h at 37°C and 5% CO_2_. (E) To analyze virus production, the drops were broken and pooled, and cells and/or viral supernatant were recovered. (F) Cells and virus from drop and bulk infections were analyzed using LIVE/DEAD staining to determine cell viability, RT-qPCR to determine viral genome copy number, and plaque assays to determine viral infectivity.

A schematic of the virus infection workflow comparing drops to bulk is outlined in Fig. 1. Naive cells were infected with IAV at an MOI of 0.1 prior to encapsulation such that most cells were infected with a single infectious virus particle (Fig. 1A). Following inoculation, cells were dissociated from the tissue culture plates and processed for encapsulation into 100-μm drops (Fig. 1B), while bulk samples were replated (Fig. 1D). Both encapsulated and bulk cells were similarly processed to ensure that trypsinization from the tissue culture plates and subsequent centrifugation and washing did not interfere with infection kinetics. A 0 h bulk and drop sample was obtained immediately following replating or encapsulation. The remaining bulk and drop samples were incubated for 24 h at 37°C to allow for a minimum of one round of virus replication (Fig. 1C and D). At 24 hours post infection (hpi), viral supernatant was collected from both the bulk and drop samples for analysis of viral genome copies and infectious progeny via reverse transcriptase quantitative PCR (RT-qPCR) and plaque assay. To sample viral supernatant from encapsulated infections, the drops were placed in the freezer and, after thawing, treated with 20% 1H,1H,2H,2H-perfluoro-1-octanol (PFO) in HFE7500 to break the emulsions and release the cells and viral supernatant for collection (Fig. 1E). To assess cell viability, drops were not frozen and were broken with 20% PFO in HFE7500 only. We then compared cell viability and number of viral genomes and infectious progeny from different cell lines maintained in bulk culture and encapsulated in drops (Fig. 1F).

### Validating IAV infection in different cell lines.

**FIG 2:**
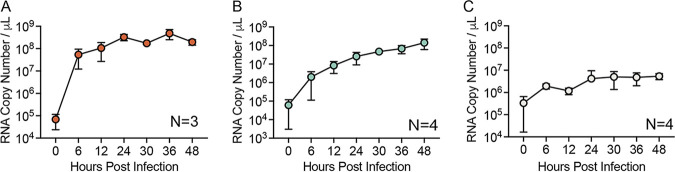
IAV infection in different cell lines. IAV genomes, as measured by RT-qPCR over 48 hpi from supernatant of infected A549 (A), MDCK (B), and siat7e (C) cells. All data represent the mean ± SD of a minimum of three independent replicates.

Before performing IAV infection in drops, IAV infection was studied in bulk using three different cell lines, A549, MDCK, and siat7e cells. Both the A549 and MDCK cells are anchorage dependent and are commonly used to propagate IAV and investigate viral infection phenotypes. The siat7e cells are a modified MDCK cell line that expresses the *siat7e* gene (ST6GalNac V), which is important for cellular adhesion, allowing the cells to grow in a suspension culture, and they are distinctive from the standard MDCK-Siat1 cells used commonly for IAV production (28, 29). They were developed to simplify cell culture-based IAV vaccine production (28, 29). The siat7e cells are anchorage independent and were tested for their potentially more favorable compatibility to drop encapsulation (4, 7). IAV production was compared between the different cell lines to determine the kinetics of viral infection in a bulk infection. The cells were infected at an MOI of 0.1, and viral RNA was measured from supernatant at 0, 6, 12, 24, 30, 36, and 48 hpi using RT-qPCR, in which the 0 h measurement represents the inoculating dose (Fig. 2A to C). A549 cells demonstrated the most robust viral RNA production during infection, with 9.5 × 10^4^ genome copies/μL at 0 hpi, which increased to 3.9 × 10^8^ genome copies/μL at 24 hpi, a 1,000-fold increase. Between 24 and 48 hpi, the amount of RNA detected fluctuated slightly but remained between 1.6 and 6.1 × 10^8^ genome copies/μL. MDCK cells demonstrated a slower increase in viral production, with 1.1 × 10^5^ genome copies/μL at 0 hpi, increasing to 3.5 × 10^7^ genome copies/μL at 24 hpi, a 100-fold increase, before reaching 1.9 × 10^8^ genome copies/μL at 48 hpi. IAV production in siat7e cells was limited with no exponential increase over the course of 48 hpi, with 6.0 × 10^5^ genome copies/μL at 0 hpi and 5.4 × 10^6^ genome copies/μL at 48 hpi. For A/California/07/2009, peak hemagglutinin (HA) titers in siat7e cells were previously observed at 24 hpi, although 50% tissue culture infective dose (TCID_50_) measurements showed similar kinetics to our observed viral RNA measurements, with limited increases in titers from 0 to 120 hpi, suggesting that replication and production of infectious virus in the siat7e cells is limited (29). In addition, viral growth, as measured by TCID_50_, of A/Brisbane/59/2007 IVR-148 (H1N1) and A/Uruguay/716/2007 X-175C (H3N2) in the siat7e cells was also not observed until 96 and 84 h, respectively, suggesting that there is variability in viral replication and kinetics in the siat7e cells (11). Overall, the average genomes/μL for A549, MDCK, and siat7e cells from 6 to 48 hpi, the time frame that represents active IAV production, was 2.6 × 10^8^, 6.4 × 10^7^, and 2.9 × 10^6^ genome copies/μL, respectively, demonstrating cell type-dependent differences in IAV production.

### Drop stability and cell viability during incubation.

**FIG 3:**
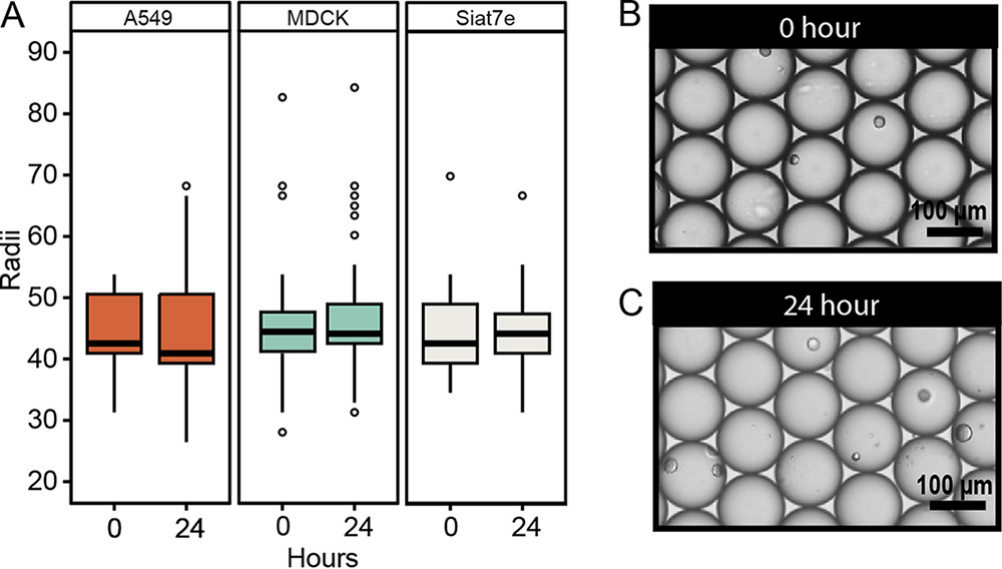
Drop stability in 100-μm microfluidic drops. (A) Drop radii (*R*) of encapsulated A549 (*n* = 4,067), MDCK (*n* = 4,235), and siat7e (*n* = 3,858) cells at 0 and 24 h. Representative images of A549 cells encapsulated in drops at 0 h (B) and 24 h (C). Scale bars, 100 μm.

To perform long-term infection studies of viral infection in drops, drops must remain stable against coalescence, and cells must remain viable to propagate virus. Therefore, drop radii and cell viability over 24 h were first quantified before performing viral infection experiments. The A549, MDCK, and siat7e cells were encapsulated in 100-μm drops and incubated for 24 h. Drop radii (*R*) were measured following encapsulation at 0 h and after incubation of all three cell lines at 24 h (Fig. 3A). For A549 cells, the average *R* at 0 h was 45.1 μm with a 95% confidence interval (CI) of 35.9 to 54.3 μm, which decreased by 0.9 μm to 44.2 μm, with a 95% CI of 0.9 to 0.7 μm after incubation for 24 h. For MDCK cells, the average *R* at 0 h was 44.6 μm with a 95% CI of 35.3 to 53.9 μm, which increased by 1.3 μm to 45.9 μm with a 95% CI of 1.2 to 1.5 μm after incubation for 24 h. For the siat7e cells, the average *R* at 0 h was 44.8 μm with a 95% CI of 35.5 to 54.2 μm, which increased by 0.7 μm to 45.5 μm with a 95% CI of 0.6 to 0.9 μm after incubation for 24 h. A linear mixed-effects model was used to determine if there was a significant change in *R* as a function of the cell type or time point postencapsulation. The change in *R* from 0 to 24 h following encapsulation of either A549 or MDCK cells was considered significantly different (*P* ≤ 0.001 for both), while the change in drop *R* from encapsulated siat7e cells was not significant (*P* = 0.323). However, since the changes in *R* for both cell lines are less than 3% of the original drop sizes and correspondingly less than the camera resolution of 1.6 μm/pixel, these data indicate that cell incubation does not have a meaningful impact on changes in drop stability as measured by *R*. Representative images of encapsulated A549 cells at 0 h (Fig. 3B) and 24 h (Fig. 3C) demonstrate this drop stability.

**FIG 4:**
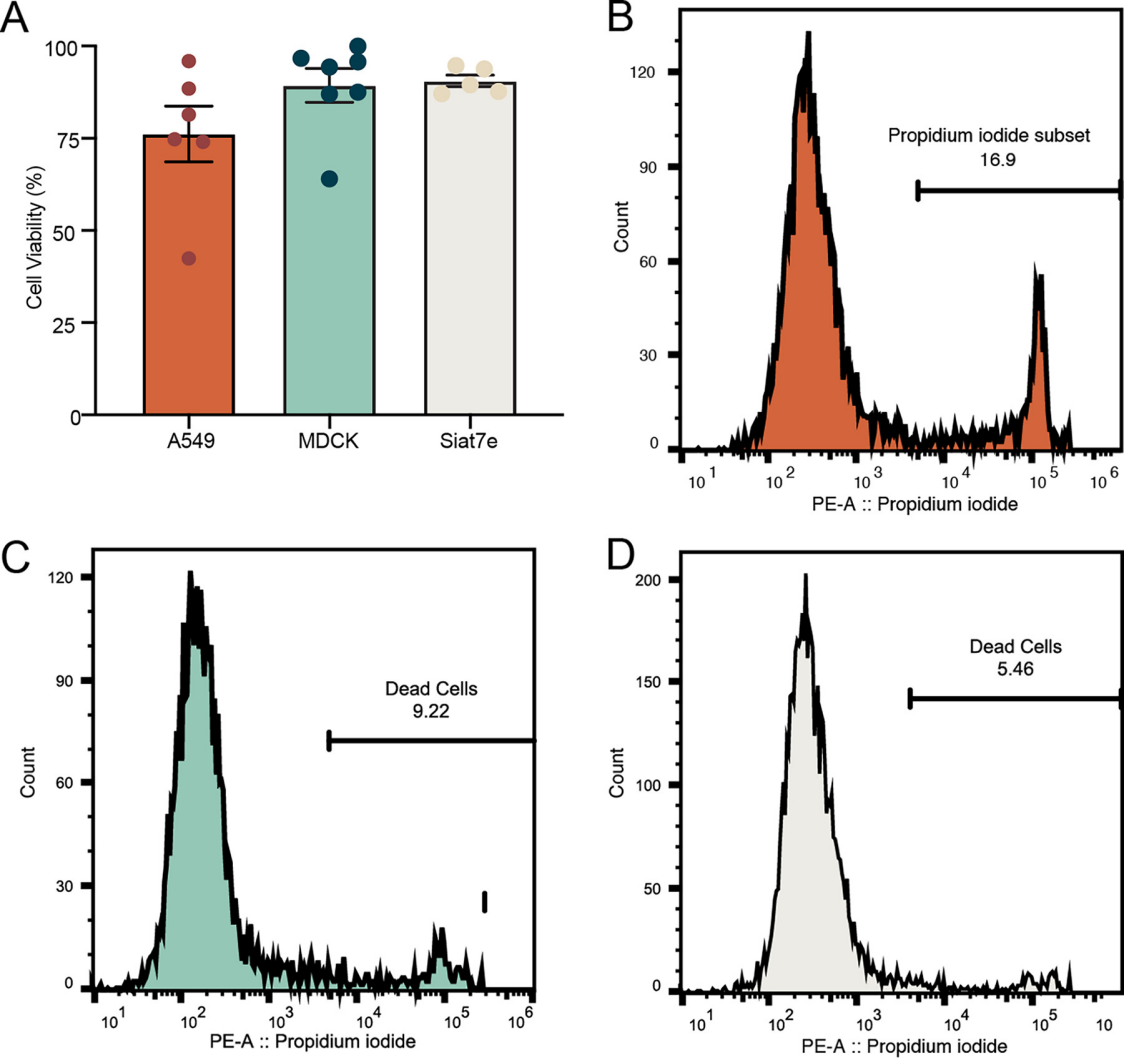
Cell viability in 100-μm microfluidic drops. (A) A549, MDCK, and siat7e cell viability at 24 h postencapsulation as determined by trypan blue staining, represented by the mean ± standard error of the mean (SEM). Points represent individual experiments. (B to D) FACS analysis of cell viability for A549 (B), MDCK (C), and siat7e (D) cells. The line on the histogram indicates the gating strategy to identify apoptotic cells.

To assess cell viability, drops containing cells were broken after 24 h of incubation, and the aqueous layer which contained the cells was collected and analyzed with a colorimetric cell viability stain and by FACS analysis. Following staining and counts of the cells using a hemacytometer, each of the three cell lines showed cell viability over 75%. Average live cell viability percentages were 76.2 ± 7.6% for A549 cells, 89.3 ± 4.6% for MDCK cells, and 90.6 ± 3.5% for siat7e cells. FACS analysis was used to quantify cell viability of a larger population of cells incubated in drops for 24 h (Fig. 4B to D). Cells were stained with propidium iodide to identify apoptotic cells within the population. A minimum of 10,000 cells were analyzed. The percentage of viable A549 cells following drop incubation was 83.1%. For MDCK cells, the percentage of viable cells was 90.7%, while viability of the siat7e cells was 94.5%. For all three cell lines, the percentage of viable cells determined by FACS was in the margin of error for the values determined by trypan blue staining.

The high cell viability observed in the siat7e cells was expected due to the cells being nonadherent with fewer physiological changes when encapsulated within drops. While the viability of the A549 cells was less than that observed in the MDCK and siat7e cells in drops, there was not a significant difference. We hypothesize that the differences observed in viability between the adherent A549 and MDCK cells were due to inherent differences between the cells themselves. MDCK cells are more prone to overgrowing and detaching from tissue culture flasks. They do not display strong contact inhibition and may be slightly better suited for drop encapsulation. In comparison, A549 cells demonstrate strong contact inhibition and rarely detach from tissue culture flasks. These inherent differences may impact cell viability following encapsulation into microfluidic drops.

### Cell loading in microfluidic drops.

**FIG 5:**
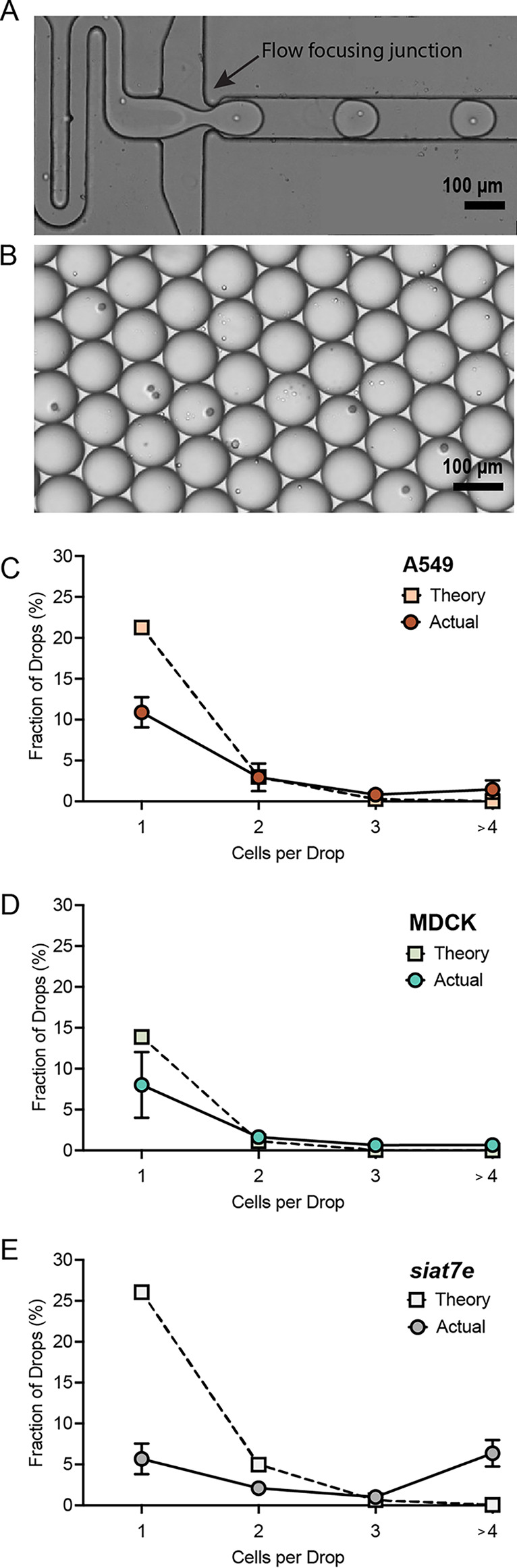
Cell encapsulation in microfluidic drops. (A) Still image of high-speed camera footage during cell encapsulation. The image has been modified to cover lithography marks outside the channels for aesthetics. (B) Representative image of drops encapsulated onto a hemocytometer. (C) A549 cell loading as measured with the hemocytometer (red circle, *n* = 600) compared to the theoretical Poisson distribution (peach square). (D) MDCK cell loading as measured with the hemocytometer (aqua circle, *n* = 601) compared to the theoretical Poisson distribution (green square). (E) Siat7e cell loading as measured with the hemocytometer (light gray circle, *n* = 598) and compared to the theoretical Poisson distribution (white square). Data points are represented by the mean ± SD. All scale bars are 100 μm.

For single-cell studies, each drop should be loaded with one cell. The number of cells loaded into drops can be estimated using a Poisson distribution (37) as follows:

$$p\left(k,\text{λ}\right)=\frac{{\text{λ}}^{k}e^{-\text{λ}}}{k!}$$

where *p* is the probability of finding *k* cells per drop, *k* is the number of cells per drop, and λ is the mean number of cells in the volume of each drop. The λ is calculated by multiplying the starting cell density by the volume of a drop (22). Based upon Poisson statistics, loading cells at a density of 2 × 10^6^ cells/mL into 100-μm drops should result in a λ of 1.0, with 37% of drops containing no cells, 37% of drops containing one cell, 18% of drops containing two cells, 6% of drops containing three cells, and 1.9% of drops containing four or more cells. To check if the three cell lines followed the predicted Poisson distribution, cells were loaded at a density of 2 × 10^6^ cells/mL, and cell counts were made using still images of drops loaded onto a hemocytometer (Fig. 5A and B). Our calculated distribution did not follow expected Poisson statistics. The observed cell loading distributions for all three cell lines were much lower than expected. The percentages of drops that contained either 0, 1, 2, 3, or 4+ cells per drop were determined to understand how well the cell loading followed the predicted Poisson distribution. A549 cells had 10.8 ± 1.8% of total drops containing one cell from the images acquired on the hemocytometer, compared to the predicted 37% (Fig. 5C). MDCK cells had 8.0 ± 4.0% of total drops containing one cell (Fig. 5D), and siat7e cells had 5.7 ± 1.9% of total drops containing one cell on the hemocytometer (Fig. 5E). The percentage of loaded drops that contained a single cell only was also calculated. For A549 and MDCK cells, 68% and 73% of loaded drops contained a single cell, respectively (Table 1). For siat7e cells, only 38% of loaded drops contained a single cell (Table 1). With lower than expected cell loading data, we recalculated λ based on the experimentally determined loading distribution to accurately calculate the concentration of cells loaded into drops, not just added to the syringe for encapsulation. Based upon the experimental loading distribution, λ for each cell line was calculated using a Poisson mixed-effects model. The λ values correspond to starting cell concentrations that were lower than the input concentration of 2 × 10^6^ cells/mL. The A549 cells had a λ of 0.282, which corresponds to an estimated cell concentration of 2.80 × 10^5^ cells/mL. The MDCK cells had a λ of 0.164, which corresponds to an estimated cell concentration of 3.19 × 10^5^ cells/mL. The siat7e cells had a λ of 0.382 and an estimated cell concentration of 3.85 × 10^5^ cells/mL. We hypothesize that the discrepancy between the starting cell concentration and observed loading distribution was most likely due to cell settling in the syringe during loading and cell adherence to the filter upstream of the flow-focusing junction. The theoretical Poisson distributions that correspond to the calculated λ values for each cell line were determined (Fig. 5C to E). A chi-square analysis was used to evaluate how the actual distribution of cells in drops compared to the theoretical Poisson distributions. A higher *P* value indicates a lack of evidence that the measured distribution does not follow a Poisson distribution. The A549 cells had the highest *P* value of 0.70, while the MDCK cells had a *P* value of 0.48. The drops containing the siat7e cells had the lowest *P* value of 0.15, suggesting that the distribution of siat7e cells in drops was the least representative of a Poisson distribution out of the three cell lines. Overall, the siat7e cells demonstrated the greatest variability and unpredictability in cell loading. We hypothesize that this is due to the cells adhering together, which is also visible during normal cell growth in a shaker flask in which large clumps of cells arise during growth. Due to poor cell encapsulation and low IAV replication during infection, siat7e cells were excluded from further analysis.

**TABLE 1:** Percentage of cells/drop in loaded drops containing cells

Cell type	% 1 cell/drop	% 2 cells/drop	% 3 cells/drop	% 4+ cells/drop
A549	67.6	18.2	5.1	9.1
MDCK	72.9	14.9	6.0	6.2
siat7e	36.5	15.5	6.2	41.8

### IAV propagation in microfluidic drops.

**FIG 6:**
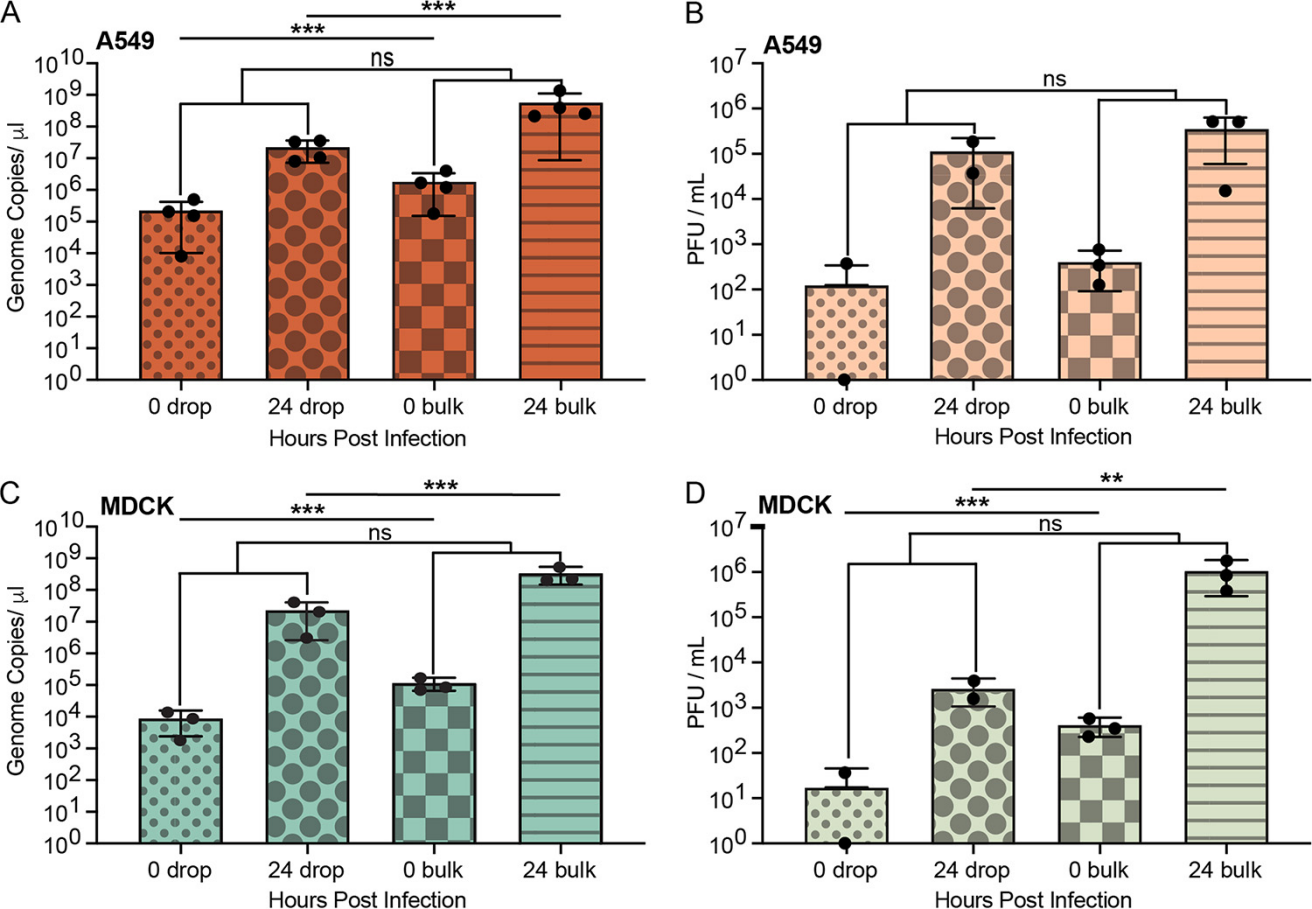
Comparison of drop and bulk IAV infections in A549 and MDCK cells. (A) RNA copy number of A/California/07/2009 (H1N1) IAV in A549 cells infected at an MOI of 0.1 at 0 hpi in drops (red small dots), 24 hpi in drops (red large dots), 0 hpi in bulk (red squares), and 24 hpi in bulk (red stripe). (B) Infectious virions (PFU/mL) released from A549 cells at 0 hpi in drops (peach small dots), 24 hpi in drops (peach large dots), 0 hpi in bulk (peach squares), and 24 hpi in bulk (peach stripe). (C) RNA copy number of A/California/07/2009 (H1N1) IAV in MDCK cells infected at an MOI of 0.1 at 0 hpi in drops (aqua small dots), 24 hpi in drops (aqua large dots), 0 hpi in bulk (aqua squares), and 24 hpi in bulk (aqua stripe). (D) Infectious virions (PFU/mL) released from MDCK cells 0 hpi in drops (green small dots), 24 hpi in drops (green large dots), 0 hpi in bulk (green squares), and 24 hpi in bulk (green stripe). All data represented as mean ± the SD with a minimum of three independent experiments. Significance is based upon a two-tailed *t* test (**, *P* < 0.01; ***, *P* < 0.001; ns, not significant).

Following analysis of cell encapsulation and viability of A549 and MDCK cells, IAV propagation was compared between A549 and MDCK cells in bulk and in drops. A549 and MDCK cells were infected at an MOI of 0.1 with the A/California/07/2009 (H1N1) IAV strain. To quantify the increase in virus genomes and infectious progeny for both bulk and drop infections at 0 and 24 hpi, viral RNA was measured using RT-qPCR, and infectious virus output was measured using plaque assay. Measurements were taken at 0 and 24 hpi to limit detection of secondary spread within bulk cultures, as a single round of replication occurs between 16 and 24 hpi (38). For A549 cells, the number of genome copies/μL in drops, as measured by M gene RNA copy number using RT-qPCR, was 2.3 × 10^5^ genome copies/μL at 0 h and increased to 2.3 × 10^7^ genome copies/μL at 24 h (Fig. 6A). Bulk infections of A549 cells had a comparable increase, with 1.8 × 10^6^ genome copies at 0 h and 5.6 × 10^8^ genome copies/μL at 24 h (Fig. 6A). The log RNA concentrations in drops compared to bulk at 0 h and 24 h were significantly different (*P* = 0.0006 and *P* = 4.3E-09, respectively), which we hypothesize is due to a lower number of total cells loaded into drops during encapsulation as described in “Cell loading in microfluidic drops” than cells seeded on tissue culture plates. While this was not seen in the analysis of infectious virus (PFU/mL) for A549 cells (*P* = 0.31 for 0 h and *P* = 0.71 for 24 h) (Fig. 6B), it was observed for both RNA (*P* = 7.2E-05 for 0 h and *P* = 0.0002 for 24 h) and PFU/mL for MDCK cells (Fig. 6C and D). However, the log difference of viral genomes produced by cells from 0 to 24 h in drops compared to cells in bulk was not significant (*P = *0.057) and suggests that overall virus production was not impacted by cell encapsulation. Recovery of infectious virus from A549 cells over the same 24-h incubation period was not significantly different (*P* = 0.84) between IAV infection in drops and bulk culture with 1.1 × 10^5^ PFU/mL and 3.6 × 10^5^ PFU/mL recovered at 24 hpi, respectively (Fig. 6B).

For MDCK cells, the number of genome copies in drops was 8.9 × 10^3^ genome copies/μL at 0 h and increased to 2.3 × 10^7^ genome copies/μL at 24 h, whereas bulk infections of MDCK cells increased from 1.2 × 10^5^ genome copies/μL at 0 h and 3.4 × 10^8^ genome copies/μL at 24 h (Fig. 6C). Similar to A549 cells, the overall log difference of viral genomes produced by cells from 0 to 24 h was not significantly different (*P* = 0.6) between drop and bulk infection. Recovery of infectious virus from MDCK cells over 24 h in drops was 2.6 × 10^3^ PFU/mL, while bulk infections produced 1.0 × 10^6^ PFU/mL (Fig. 6D). In contrast to A549 cells, the log PFU/mL concentrations measured in MDCK cells in drops compared to bulk at 0 h and 24 h were significantly different (*P* = 0.0004 and *P* = 0.003, respectively); however, this did not translate into a significant difference in the change in log PFU/mL concentration from 0 to 24 h between drop and bulk infections. The PFU-to-genome ratio for MDCK cells was 1 PFU per 5.81 × 10^3^ genomes in drops and 1 PFU per 3.24 × 10^2^ genomes in bulk. In comparison, for A549 cells, the PFU-to-genome ratio results in 1 PFU per 2.97 × 10^2^ genomes in drops and 1 PFU per 1.63 × 10^3^ genomes in bulk.

## DISCUSSION

The drop-based microfluidic methods developed in this work will enable future studies of single-cell IAV infections using multiple cell lines. The methods expand the application of drop-based microfluidics from the nonenveloped, positive-sense MNV-1 (4, 7, 8) to include the enveloped and negative-sense IAV, thus broadening the scope of single-cell virology assays. Additionally, this work can serve as a blueprint for testing, comparing, and implementing drop-based microfluidics methods in future single-cell studies. With the recent interest and development of single-cell omic technologies for studying viral infections (13–15, 20, 30–36), we expect this work to further expand capabilities and increase applications of single-cell technologies in virology.

To successfully perform in drop infections, standard cell lines used for propagating viruses need to remain viable, and this depends upon both drop size and cell type. Previous studies exploring viability of cells in drops found that smaller drop sizes resulted in low levels of cell viability, even after a couple of hours, which is most likely due to a lack of enough nutrients or buildup of waste products within the drops (39). Here, we demonstrate that 100-μm-diameter drops yielded high cell viability and drop stability following 24 h of incubation. Cell viability of A549, MDCK, and siat7e cells in 100-μm drops remained high (>75%), and the average drop radii changed by less than 3%. High cell viability was expected from the siat7e suspension cell line, which was found to have a mean of 91%. Though more variable between experimental replicates compared to the siat7e cells, the adherent A549 and MDCK cell lines still demonstrated high cell viability (Fig. 3D).

To analyze the distribution of cells in drops, we used a hemocytometer to determine cell loading distributions. A concentration of 2 × 10^6^ cells/mL was calculated to obtain a maximum number of 100-μm diameter drops that contain a single cell based upon Poisson loading. However, throughout our experiments, cells settled in the syringes and attached to the aqueous-stream filter on the microfluidic drop maker, lowering the number of cells being encapsulated. Therefore, the average number of cells per drop volume, as described by λ in our experiments, corresponded to a lower cell concentration on the order of 10^5^ cells/mL. One option to reduce cell settling is the use of density-matched medium cells during encapsulation with a biologically inert polymer, such as OptiPrep (40). This would decrease the settling velocity of a cell as estimated using Stokes’ law (40). However, cell media components would have to be further optimized, as increases in OptiPrep concentration also decrease cell viability (40). Alternatively, a larger vessel in which cells are constantly stirred and then pneumatically transferred into the microfluidic device would also decrease cell settling. Finally, cell adherence to the upstream filter was caused by clumps of cells, likely free DNA and cell debris released during cell lysis. A reduction in shear stress that the cells experience by using gentle pipetting or syringe loading would aid in reducing cell debris. Cell adherence to device structures can be reduced by eliminating the filter structures or increasing the gaps between the pillars within the microfluidic device.

While cell settling impacted the cell loading concentration, the majority of our loaded drops still contained a single cell. Surprisingly, the suspension siat7e cell line had the largest variation from the expected Poisson distribution, with more drops having four or more cells/drop than was observed for A549 or MDCK cells. We hypothesize that this large variation was due to the siat7e cells adhering to each other when cultured in a spinner flask and during infection prior to cell loading. To improve cell loading, a higher concentration of cells can be used, although previous analysis of 2 × 10^6^, 4 × 10^6^, and 8 × 10^6^ cells/mL in 50-μm drops had similar loading distributions (4). Thus, we conclude that cell loading depends on the cell type, with adherent A549 and MDCK cells resulting in a majority of single cells loaded into drops compared to the suspension siat7e cells.

Following our success in encapsulation and culturing multiple cell lines within microfluidic drops, we investigated whether infected cells produce virus following encapsulation compared to bulk studies. Due to the highly variable cell loading and low virus production observed in the siat7e cells, only the A549 and MDCK cells were utilized for drop infection experiments. Measurements of viral RNA via RT-qPCR and infectious progeny via plaque assay were made at 0 and 24 h from supernatant collection from broken drops and compared to standard bulk infections on well plates. The amount of virus produced from A549 and MDCK cells between 0 and 24 h in bulk was not significantly different than the amount of virus produced from cells in drops. This demonstrates that microfluidic drops can be used to propagate IAV from individual cells and that standard adherent cell lines can be used without the need to adapt adherent cells to suspension cultures. To further evaluate single-cell kinetics of infection, multiple time points could be analyzed from infected cells in drops. One proposed method for performing this analysis is the use of fluorescent protein (FP) expression from specific viral or cellular gene targets. This would allow for continuous observations of cells in drops over time while quantifying changes in FP expression that correlate with virus replication or transcription. Time-resolved control using drops could expand the capabilities of previous work in which fluorescent proteins expressed by herpes simplex virus 1 were used to isolate infected cells at 5 hpi for downstream single-cell RNA sequencing (scRNA-seq) (14). This work lays the foundation to expand methods for investigating the heterogeneity within population dynamics at the single-cell level.

One concern regarding incubating viruses in drops is the transfer of viruses between drops and the potential loss of infectious virus upon drop breaking. The surfactant that is used to stabilize the drops is a mixture of di-block and tri-block polymers with fluorinated tails that extend into the oil phase and hydrophilic blocks that extend into the aqueous phase that are PEGylated (26). Polyethylene glycol (PEG) prevents most biological materials from interacting with the drop interface and diffusing between neighboring drops. Small molecules (water, amino acids, ions, and some antibiotics) have been known to diffuse between neighboring drops (41–43); however, under our drop conditions, we empirically observe that viruses do not cross the drop interface.

### Conclusions.

Analysis of individually infected cells has revealed the complexity and heterogeneity of viral infections (9–11, 20). The implementation of current omics technologies in these systems has demonstrated that viral infections are as heterogeneous and complex at the single-cell level as they are at the systems level (23–25). The application of new technologies for the study of virology can elucidate how individual host cells influence viral evolution and respond to infection through the innate and adaptive immune systems. Considering the current COVID-19 pandemic and the emergence of multiple variants within the viral population, it is critical to expand our ability to study of how these variants arise, how they operate under selective pressures, and how they impact transmission and virulence (44). Single-cell studies can be limited by low-throughput analysis, reliance on expensive cell-sorting machinery, and genetic manipulation of the virus itself. The use of drop-based microfluidics can expand single-cell studies, offering the ability to perform high-throughput analysis of individually infected cells (5, 21, 31). Advantageously, these applications can be adapted to many laboratories with minimal investment. The ability to expand single-cell analysis using drop-based microfluidics has the potential to revolutionize the field of virology.

## MATERIALS AND METHODS

### Cells and viruses.

Human alveolar epithelial cells (A549), Madin-Darby canine kidney cells (MDCK), and MDCK plus human siat7e gene (siat7e) cells were obtained from ATCC (CCL-185, CCL-34) and Joseph Shiloach (NIH), respectively (28, 29). A549 cells were propagated in Hams F-12 (Corning) medium supplemented with 10% fetal bovine serum (HyClone) and 1× penicillin-streptomycin (Fisher Scientific). MDCK and siat7e cells were propagated in Dulbecco’s modified Eagle medium (DMEM; Corning) supplemented with 10% fetal bovine serum (HyClone) and 1× penicillin-streptomycin. The IAV virus strain A/California/07/2009 (H1N1) was obtained from Christopher Brooke (University of Illinois Urbana-Champaign [UIUC]). Stocks of A/California/07/2009 were propagated, and we determined their titer on MDCK cells.

### Microfluidic device fabrication.

Microfluidic devices for drop making were fabricated by patterning SU-8 photoresist (Microchem; catalog no. SU-8 50) on silicon wafers (University Wafer; ID no. 447; test grade) to create 100-μm-tall and 100-μm-wide channels. Polydimethylsiloxane (PDMS) (Sylgard 184) was poured onto the wafers at a 10:1 mass ratio of polymer to cross-linking agent according to standard photolithography techniques (45). Air was purged from the uncured PDMS by placing the filled mold in a vacuum chamber for at least 1 h. The PDMS was cured in an oven at 55°C for 24 h, and ports were punched into the PDMS slab with a 0.75-mm-inside-diameter biopsy specimen punch (EMS Rapid-Core; Electron Microscopy Sciences). The PDMS slab was bonded to a 2-in. by 3-in. glass slide (VWR; microslides, catalog no. 48382-179) after plasma treatment (Harrick Plasma; catalog no. PDC-001) for 60 s at high power (30 W) and 700 mtorr oxygen pressure. The drop-making devices were made hydrophobic by flowing Aquapel (Pittsburgh Glass Works) through the channels, followed by blowing the channels with air filtered through a GVS Abluo 25-mm 0.2-μm filter (Fisher Scientific) before baking the devices in an oven at 55°C for 1 h.

### Cell encapsulation in drops.

Cells were seeded onto T25 flasks or 6-well plates at a density of 1 × 10^5^ cells/cm^2^ and incubated overnight. Cells were prepared for encapsulation by washing 2× with phosphate-buffered saline (PBS) followed by trypsinization to remove cells from the surface of the flasks or plates. The appropriate culture media containing 10% fetal bovine serum (FBS) were added to the cells to neutralize trypsinization, and cells were collected and centrifuged at 500 × *g* for 5 min. The cell pellet was gently washed with 3 to 5 mL of PBS and centrifuged a second time at 500 × *g* for 5 min. The cell pellets were gently resuspended in FBS-free media to reach 2 × 10^6^ cells/mL in their designated media. We placed 10 μL of cells on a hemocytometer to visualize and confirm cell concentration and verify the presence of mostly single cells. Cells were loaded into a 3-mL syringe (BD) for injection onto a flow-focusing microfluidic device for encapsulation into 100-μm drops (~523 pL) (46). Drops were stabilized in a fluorinated HFE7500 oil (3 M) continuous phase with a 1.5% (wt/wt) solution of PEG-PFPE_2_-based surfactant (RAN Biotechnologies; 008-FluoroSurfactant). The fluids were transferred into the microfluidic device with syringe pumps (New Era NE-1000) controlled by a custom LabVIEW (2015) program at flow rates of 1,000 μL/h for the aqueous phase and 3,000 μL/h for the oil phase. A 1,500-μL total volume consisting of 375 μL of drops containing cells and 1,125 μL of oil was collected in a 2-mL microcentrifuge tube (Eppendorf). Following encapsulation, drops containing cells were incubated in an open microcentrifuge tube covered with parafilm at 37°C with 95% relative humidity and 5% CO_2_. Cells were released from drops by removing most of the oil phase and then adding 1 mL of a 20% wt/wt 1H,1H,2H,2H-perfluoro-1-octanol (PFO) in HFE7500 oil to break the emulsion, followed by vortexing. Centrifugation at 500 × *g* for 5 min was used to separate the aqueous cell suspension from the oil phase to isolate cells for further analysis.

### Drop measurements.

Drop radii were measured after imaging a drop monolayer on a hemocytometer with a charge-coupled-device (CCD) camera (Flir Grasshopper3) on an inverted microscope (Nikon Ti-U). A monolayer of drops with minimal packing density is viewed using a hemocytometer so that the drops remained spherical for image analysis. A custom MATLAB script was used to determine the drop radii, *R*, at 0 h and 24 h after the cells were incubated. Six images of drops, with a minimum of 300 drops per image per time point, were analyzed. A data set containing *R* values at 0 and 24 h post encapsulation, the encapsulated cell line, and experimental dates was created to analyze changes in *R*. A linear mixed-effects model, with the experimental date as a random factor, was used to analyze the contributions of cell type and time point to the data set.

### Cell loading and viability.

The distribution of cells per drop was determined using still images of a drop monolayer on a hemocytometer as described in “Drop measurements.” These distributions were fit to a Poisson mixed-effects model with cell lines as the fixed effect and date as the random effect (47, 48). We used a Pearson chi-square goodness-of-fit test to determine whether the observed data did not fit the theoretical distribution. Following encapsulation, drops were incubated at 37°C with 95% relative humidity and 5% CO_2_. Cells were released from drops as previously described. The cells in the aqueous phase were collected and pelleted at 1,500 × *g* for 5 min. The cell pellet was resuspended in 200 μL PBS. Cells were diluted 1:5 in PBS with 10% trypan blue stain for a final volume of 50 μL to determine cell viability. An unpaired *t* test was used to determine if cell viability was significantly different between the cell lines. Flow cytometry (FACS) analysis was performed on cells isolated as described above and resuspended in PBS with 2% FBS and 5 μg/mL propidium iodide (Sigma-Aldrich; catalog no. 537060) to stain apoptotic cells. Samples were analyzed with a BD LSRFortessa flow cytometer (BD Biosciences) and FlowJo software (Tree Star).

### IAV infection of adherent cells (A549 and MDCK).

Cells were seeded onto a 6-well plate at a concentration of 1 × 10^6^ cells/well and infected with IAV H1N1 at an MOI of 0.1 based upon plaque assay titering in infection media. The infection media consisted of Hams F-12 or DMEM supplemented with 1 mM HEPES (HyClone), 1× penicillin-streptomycin, 0.1% bovine serum albumin (BSA) (MP Biomedical), and 1 μg/mL of tosylsulfonyl phenylalanyl chloromethyl ketone (TPCK)-trypsin (Worthington Biomedical) (49). Briefly, cells were washed with 1× PBS (Corning) and incubated with 200 μL of virus inoculum for 1 h. The inoculum was removed and replaced with 1.5 mL fresh infection medium for another 1 h of incubation. Infection medium was removed, cells were washed with PBS, and the infected cells were detached from the plate by trypsinization. Infected cells were processed as described in “Cell encapsulation in drops” and resuspended in encapsulation media containing either Hams F-12 or DMEM and 1 mM HEPES, 1× penicillin-streptomycin, and 0.1% BSA. The infected cell suspension was split into two populations, with one population replated as a bulk control and the other encapsulated in 100-μm drops. Bulk and drop infections were incubated at 37°C with 95% relative humidity and 5% CO_2_ and frozen at −20°C at 0 and 24 h postinfection (hpi).

### IAV infection of suspension siat7e cells.

To infect the siat7e suspension cells at an MOI of 0.1 based upon plaque assay titering, 1 × 10^7^ cells were pelleted at 500 × *g* for 5 min in a 50-mL conical centrifuge tube and then resuspended in 200 μL of virus inoculum in DMEM supplemented with 1 mM HEPES (HyClone), 1× penicillin-streptomycin, 0.1% BSA, and 1 μg/mL of TPCK-trypsin (49). The 50-mL tube containing the cells and virus inoculum was shaken at 90 rpm for 1 h. The cells were pelleted at 500 × *g* for 5 min, and the inoculum was removed and replaced with fresh DMEM infection media (10 mL) for bulk infections or DMEM encapsulation media as described in “IAV infection of adherent cells (A549 and MDCK).” Infected cells were either encapsulated in 100-μm drops or replated as a bulk control. For bulk infections, a 1-mL volume of cells was added to each well of a 6-well plate (2 total) and placed on the shaker. Encapsulated cells were processed as described in “Cell encapsulation in drops.” Bulk and drop infections were incubated at 37°C with 95% relative humidity and 5% CO_2_ and frozen at −20°C at 0 and 24 hpi.

### M gene abundance by RT-qPCR.

RNA was extracted from cell suspensions using the Qiagen QIAamp viral RNA minikit. RNA copy number of the IAV matrix protein gene (M gene) was determined using a TaqMan RT-qPCR assay (5). Amplification primer sequence was originally described by Shu et al. (50) as follows: M gene forward, 5′-GACCRATCCTGTCACCTCTGAC-3′, and M gene reverse, 5′-AGGGCATTCTGGACAAATCGTCTA-3′. The sequence of the M gene TaqMan probe was 5′-/FAM/TGCAGTCCTCGCTCACTGGGCACG/BHQ1/-3′. Working stocks of the primers and probe (Eurofins Operon) were prepared at 25 μM and 10 μM, respectively, for use in the RT-qPCR. Samples were amplified using a SuperScript III Platinum one-step RT-qPCR kit (Invitrogen; catalog no. 11732-020) with a final reaction volume of 25 μL. Each reaction mix contained 400 nM M gene forward and reverse primers, 200 nM M gene TaqMan probe, 0.05 μM ROX reference dye, 5 U/μL Superase RNase inhibitor (Invitrogen; catalog no. AM2694), and 2.5 μL of RNA template. Thermocycling was performed in an RT-qPCR machine (QuantStudio 3; Applied Biosystems) with the following cycling conditions: 1 cycle for 30 min at 60°C, 1 cycle for 2 min at 95°C, and 40 cycles between 15 s at 95°C and 1 min at 60°C.

### Plaque assay.

Postinfection IAV titers were determined by plaque assay on MDCK cells seeded in 6-well plates at 1 × 10^6^ cells per well. For drop infections, the viral supernatants were sampled from broken drops at 0 and 24 hpi. For bulk infections, viral supernatants were sampled from the tissue culture plate wells at 0 and 24 hpi. The supernatants were serially diluted in DMEM media with 1 mM HEPES (HyClone), 1× penicillin-streptomycin, 0.1% BSA, and 2 μg/mL of TPCK-trypsin. Overlay medium consisting of 2× MEM with 2× penicillin-streptomycin, 1 mM HEPES, 2 μg/mL of TPCK, and 3% carboxymethyl cellulose (MP Biomedical) with 0.2 mg/mL DEAE-dextran (MP Biomedical) was added, and well plates were incubated at 37°C for 4 days. Plaques were fixed with 10% buffered formalin (Fisher), washed with deionized (DI) water, and stained with 0.5% crystal violet (Thermo Fisher Scientific) for visualization.
